# Rhizospheric *Lactobacillus plantarum* (*Lactiplantibacillus plantarum*) strains exhibit bile salt hydrolysis, hypocholestrolemic and probiotic capabilities in vitro

**DOI:** 10.1038/s41598-021-94776-3

**Published:** 2021-07-27

**Authors:** Neelja Singhal, Nambram Somendro Singh, Shilpa Mohanty, Manish Kumar, Jugsharan Singh Virdi

**Affiliations:** 1 Department of Biophysics, University of Delhi South Campus, New Delhi, India; 2 Department of Microbiology, University of Delhi South Campus, New Delhi, India

**Keywords:** Biotechnology, Microbiology

## Abstract

*Lactobacillus plantarum* (renamed as *Lactiplantibacillus plantarum*) has been isolated from many sources but very rarely from rhizospheric soil. This is the first report on isolation and assessment of probiotic capabilities of *L. plantarum* strains isolated from rhizospheric soil. The isolates were confirmed by 16S rRNA gene sequencing and named as NS14, NS16 and NGG. All the isolates were evaluated for bile salt hydrolysis, hypocholestrolemic potential and probiotic attributes. Our results indicated that all the strains harboured *bsh* and showed in vitro cholesterol assimilation capabilities which increased when bile salts were also present in the culture medium. Also, all the strains remained viable at high temperatures and in the presence of NaCl, lysozyme, simulated gastric juice, bile salts and, exhibited auto- and co-aggregation capabilities. Additionally, *L. plantarum* strain NS14 survived in the presence of phenols, acidic environment (pH 2–3) and was resistant to many clinically relevant antibiotics. Since, *L. plantarum* NS14 exhibited most of the desirable and essential characteristics of a probiotic it should be further investigated as a potent probiotic with an additional benefit as a hypocholesterolemic biotherapeutic. Moreover, rhizosphere can be explored as a useful ecological niche for isolating microorganisms with biotechnological and probiotic potential.

## Introduction

According to the World Health Organization (WHO) cardiovascular diseases result in the death of ~ 30% of the world population. It is estimated that by the end of the year 2030, around 23.3 million people would suffer from cardiovascular diseases^1^. The primary reason underlying cardiovascular diseases in human population is the elevated level of serum cholesterol. Although, dietary interventions and drug therapy are useful in reducing the serum cholesterol, several side effects are associated with drug therapy^2^. In recent years, several researchers have demonstrated that probiotics, especially strains of *Lactobacillus* and *Bifidobacterium* were effective in reducing the levels of cholesterol^3,4^. Probiotics are viable microbes whose consumption is associated with several health benefits. Probiotics might lower the serum cholesterol levels by various mechanisms like, assimilating cholesterol in the bacterial cell membranes, deconjugating the bile salts by producing enzymes like bile salt hydrolases (BSH) and converting the cholesterol into coprostanol, etc^5–7^. Several studies have indicated that BSH activity improves the adhesion ability of lactobacilli in the gastrointestinal tract^8^ and, organisms which cannot deconjugate bile salts show a reduced capability to remove cholesterol from the culture medium. Thus, BSH activity is an essential attribute for selecting novel probiotic strains with cholesterol-lowering potential^9^.

Bacteria which qualify as probiotics include several genera of lactic acid producing bacteria like, *Lactobacillus*, *Lactococcus*, *Streptococcus* spp. etc. The genus *Lactobacillus* with more than 200 species is the largest group of lactic acid bacteria (LAB). *Lactobacillus plantarum* (recently reclassified as *Lactiplantibacillus plantarum*)^10^ is the most versatile and metabolically diverse *Lactobacillus* sp. which has been widely used in industrial fermentation for production of fermented food products like cheese, kefir, sauerkraut, etc^11,12^. *L. plantarum* has been associated with a documented history of usage in food^13^. Additionally, it has been conferred the “generally recognized as safe” (GRAS) status by the US Food and Drug Administration (US FDA) and, the “qualified presumption of safety” (QPS) status by the European Food Safety Authorities (EFSA)^14^. Several strains of *L. plantarum* have demonstrated antibacterial and antifungal properties and many probiotic formulations based on *L. plantarum* strains are available in the market^15,16^. *L. plantarum* have been isolated from diverse sources like gastrointestinal, vaginal and urogenital tracts^17–19^, dairy products^20^, vegetables^21^ etc.

An earlier study reported the biotechnological properties of *L. plantarum* isolated from rhizosphere of olive trees and desert truffles^22^. However, to the best of our knowledge, the BSH activity, cholesterol assimilation and probiotic attributes of *L. plantarum* isolated from rhizosphere have not been reported. BSH activity has been rarely reported from bacteria isolated from environments where bile salts are usually absent^23–26^. Rhizospheric soil is an important ecological site for identification of probiotics because there is a competition among the resident microbes for the growth factors available in rhizosphere. Thus, rhizospheric bacteria evolve with plant pathogenic bacteria and fungi, which enhance their competence for survival in the microbe-rich human GI tract. This is the first report about isolation and evaluation of probiotic characteristics of *L. plantarum* isolated from rhizospheric soils collected from different sites located in India.

## Results

### Molecular identification of *L. plantarum* strains

Of the ten samples isolated from rhizospheric soils, three yielded pure cultures which were presumptively identified as LAB and named by us in our laboratory as NS14, NS16 and NGG. These were confirmed as *L. plantarum* based on 16S rRNA gene sequencing and BLAST-based homology search. The GenBank accession numbers of 16S rRNA gene sequences of NS14, NS16 and NGG were MW136055-57, respectively.

### BSH activity and *bsh* gene

**Figure 1:**
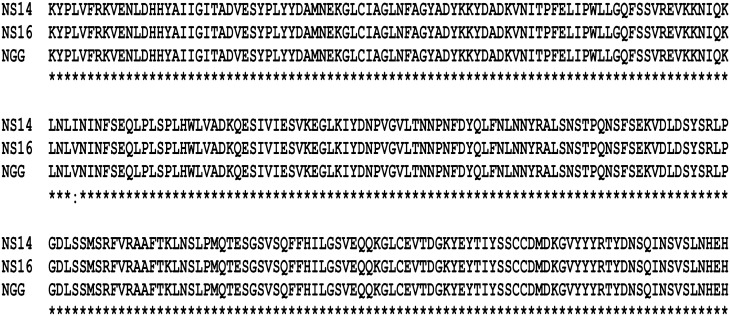
Multiple sequence alignment of amino acid sequences of bile salt hydrolases (BSH) of *L. plantarum* strains NS14, NS16 and NGG.

All the strains exhibited a white zone of precipitation (an opaque halo with a silvery shine) around their respective wells, indicating the production of BSH. In all the strains, PCR-amplification with the *bsh* primers (BF and BR) resulted in the desired amplicon of 913 bp. Gel purification; gene sequencing and BLAST analysis confirmed that the PCR amplicons encoded the *bsh* of *L. plantarum.* The results of multiple sequence alignment of BSH proteins revealed that the amino acid sequences were identical, except a variation from V to I at amino acid position 92 in *L. plantarum* strain NS14 (Fig. 1).

### Cholesterol assimilation

All the *L. plantarum* strains assimilated about 50% of the cholesterol from the medium, which improved further when bile salts were also present in the culture medium. *L. plantarum* strain NGG assimilated 48.84% cholesterol, while both NS14 and NS16 assimilated 60% cholesterol from the medium. In the presence of the bile salts, both NS16 and NGG assimilated 74% cholesterol from the medium while NS14 assimilated 92% cholesterol (Table 1). However, cholesterol assimilation capability of the strains were not statistically different from each other, (*p* > 0.05).

**Table 1:** Cholesterol removal capability of *L. plantarum* strains in cholesterol containing medium with and without bile salts.

*L. plantarum* strain	Mean cholesterol removal percentage (%) ± SD	
	Cholesterol only	Bile salts + cholesterol
NS14	60.52 ± 0.13	92.20 ± 0.60
NS16	60.40 ± 0.14	72.21 ± 0.15
NGG	48.84 ± 0.60	72.21 ± 0.16

The mean of three values ± SD are presented.

### Tolerance to temperature, salt and lysozyme

All the three *L. plantarum* strains thrived in temperatures ranging between 28 and 45 °C however, optimum growth was observed at 37 °C. Similarly, all the strains survived in MRS broth containing increasing concentrations of NaCl. All the *L. plantarum* isolates were lysozyme tolerant, showing 80% survival after 2 h of incubation in culture medium containing lysozyme (results not shown).

### Tolerance to bile salts, low pH and simulated gastric juice

All the *L. plantarum* strains survived when the bile salt concentrations were lower or equal to 0.5% even after 2–4 h *i.e.*0.125% and 0.25% (Table 2). However, none of them survived when the concentration of the bile salts in the culture medium was 1% (results not shown).

**Table 2:** Viable cells (log_10_ cfu/ml) of *L. plantarum* strains in the presence of 0.5% bile salts.

*L. plantarum* strain	Log_10_ cfu/ml		
	0 h	2 h	4 h
NS14	8.93 ± 0.04	8.86 ± 0.03	8.75 ± 0.03
NS16	8.97 ± 0.02	8.75 ± 0.04	8.73 ± 0.01
NGG	8.27 ± 0.03	8.20 ± 0.04	8.18 ± 0.02

The mean of three values ± SD are presented.

The viability of all the *L. plantarum* strains in the culture medium of pH 1.5 decreased drastically and none of the strains survived after 1–3 h. In the culture medium of pH 2, the viabilities of all the strains were reduced to almost half after 1–3 h. Both the *L. plantarum* strains NS14 and NS16 remained viable in the culture medium of pH 3.0 but the viability of NGG decreased, after 1–3 h (Table 3). Though the viability decreased slightly, but all the *L. plantarum* strains survived in simulated gastric juice even after 2 h and 3 h (Table 4).

**Table 3:** Viable cells (log_10_ cfu/ml) of *L. plantarum* strains in MRS broth at different pH and time intervals.

*L. plantarum* strain	pH 1.5			pH 2.0			pH 3.0		
	0 h	1 h	3 h	0 h	1 h	3 h	0 h	1 h	3 h
NS14	2.10 ± 0.03	–	–	2.10 ± 0.03	1.20 ± 0.01	1.03 ± 0.03	2.10 ± 0.03	2.31 ± 0.03	2.70 ± 0.03
NS16	3.72 ± 0.01	–	–	3.72 ± 0.01	1.57 ± 0.03	1.21 ± 0.01	3.72 ± 0.01	3.50 ± 0.02	3.70 ± 0.03
NGG	4.31 ± 0.02	–	–	4.31 ± 0.02	2.39 ± 0.02	2.10 ± 0.01	4.31 ± 0.02	4.10 ± 0.03	3.91 ± 0.03

The mean of three values ± SD are presented.

**Table 4:** Viable cells (log_10_ cfu/ml) of *L. plantarum* strains in simulated gastric juice.

*L. plantarum* strain	Viable cells (log_10_ cfu/ml)		
	0 h	2 h	3 h
NS14	2.82 ± 0.01	2.61 ± 0.01	2.30 ± 0.02
NS16	2.47 ± 0.01	2.37 ± 0.02	2.20 ± 0.01
NGG	2.60 ± 0.02	2.30 ± 0.02	2.12 ± 0.02

The mean of three values ± SD are presented.

### Auto-aggregation and co-aggregation capabilities

The relative auto-aggregation and co-aggregation percentage values of NS14 were 32.08% and 40.30%, respectively while of NGG were 21% and 20.10%, respectively. The relative auto-aggregation and co-aggregation percentage values of NS16 were the least *i.e.*9.15% and 9.05%, respectively. The relative auto-aggregation and co-aggregation percentage values of the *L. plantarum* strains are shown in the Table 5. However, the relative auto- and co-aggregation percentage values of the strains were not statistically different from each other (*p* > 0.05).

**Table 5:** Relative auto-aggregation and co-aggregation capabilities of *L. plantarum* strains.

*L. plantarum* strain	Auto-aggregation (%)	Co-aggregation with *E. coli* (%)
NS14	32.08 ± 0.04	40.30 ± 0.03
NS16	9.15 ± 0.03	9.05 ± 0.02
NGG	21 ± 0.05	20.10 ± 0.03

The mean of three values ± SD are presented.

### Phenol tolerance and antimicrobial susceptibilities

The *L. plantarum* strains NS14 and NS16 maintained their viability, while the viability of NGG reduced after 24 h in the culture medium containing 0.40% phenol (Table 6). *L. plantarum* strain NS14 was susceptible to amoxicillin but resistant to kanamycin, streptomycin, gentamycin tetracycline, erythromycin and tetracycline. *L. plantarum* strain NS16 was susceptible to amoxicillin, gentamicin, erythromycin and ciprofloxacin while it was resistant to streptomycin and tetracycline. *L. plantarum* strain NGG was susceptible to all the antibiotics but resistant to tetracycline (Table 7).

**Table 6:** Viable cells (log_10_ cfu/ml) of *L. plantarum* strains in 0.4% phenol.

*L. plantarum* strain	Viable cell count (log_10_ cfu/ml)	
	0 h	24 h
NS14	5.01 ± 0.01	5.94 ± 0.02
NS16	5.65 ± 0.06	5.18 ± 0.03
NGG	6.01 ± 0.01	4.24 ± 0.02

The mean values of each sample are presented along with ± SD.

**Table 7:** Antimicrobial susceptibilities of *L. plantarum* strains.

*L. plantarum* strain	AMX (10)	K (30)	STM (10)	GEN (10)	TET (30)	E (15)	CIP (5)
NS14	S	R	R	R	R	R	R
NS16	S	S	R	S	R	S	S
NGG	S	S	S	S	R	S	S

Amoxycillin (AMX), Kanamycin (K), Streptomycin (STM), Gentamicin (GEN), Tetracycline (TET), Erythromycin (E), Ciprofloxacin (CIP). S-sensitive, R-resistant. Numbers in parenthesis indicate concentration of antibiotic (µg/disc).

## Discussion

*Lactiplantibacillus plantarum* strains have been reportedly isolated from various plant and animal sources but very rarely from rhizospheric soil^22^. This is the first report about isolation and assessment of probiotic potential of *L. plantarum* strains isolated from rhizospheric soils which were collected from several sites located in central India. Rhizosphere is an interesting environmental niche which is enriched with root secretions and various plant-associated bacteria and fungi. Thus, it can be a good source for isolating probiotics exhibiting competence for survival in the microbe-rich human GI tract^27^. The 16S rRNA gene sequencing results confirmed that the three pure rhizospheric isolates named in our laboratory as NS14, NS16 and NGG were *L. plantarum*. These isolates were further assessed for bile salt hydrolysis, hypocholestrolemic and probiotic potential.

Since capability to hydrolyze bile salts has been recommended by the WHO as an important attribute for probiotic selection hence; all the *L. plantarum* strains were evaluated for bile salt hydrolysis and the presence of *bsh*^9^. The results of the phenotypic assay and gene sequencing confirmed that *bsh* was present in all the strains. Thus, our strains qualified an important attribute for probiotic selection. Also, the fact that *bsh* was present in *L. plantarum* strains isolated from rhizosphere is interesting because very few studies have reported the presence of BSH in bacteria isolated from environment naturally deficient of bile salts, like rhizospheric soil. An earlier study reported that BSH-positive *L. plantarum* strains show a greater adhesion capability for Caco-2 intestinal cell lines than BSH-negative strains^8^. This suggested that BSH-producing *L. plantarum* strains identified by us from the rhizospheric soil might pose a similar behaviour. Moreover, similarity in the amino acid sequences of the studied BSH might indicate that these proteins can be conserved in *L. plantarum*.

All the *L. plantarum* strains assimilated ~ 50% cholesterol from the culture medium which increased when bile salts were also present. For instance, *L. plantarum* strain NS14 showed higher cholesterol assimilation capability (92.20%) when bile salts were also present in the medium compared with the medium without bile salts (60.52%). The cholesterol assimilation capability of NS14 observed by us is much higher than that reported for *L. plantarum* strain RYPR1 (isolated from a fermented drink) which could assimilate only 59% of the cholesterol present in the culture medium^28^. However, the cholesterol assimilation capability of *L. plantarum* strain NS14 was lesser than that discerned for rhizospheric *Enterococcus faecium* LR13 which assimilated 98% cholesterol from the medium containing bile salts and 75% from the medium without bile salts^27^. This suggested that rhizospheric bacteria might be explored as appropriate probiotic candidates, if they can also qualify other criteria essential for probiotic status. Additionally, all the strains were assessed for various technological and probiotic characteristics. Our results indicated that all the *L. plantarum* strains could survive at temperatures as high as 45 °C and in salt concentrations up to 10% (w/v). Previous studies reported that *Lactobacillus* spp. isolated from food were tolerant to only 1–6% salt concentrations, while *L. plantarum* strain 8RA 3-pl B-RKM 0015 isolated from a commercial preparation was reportedly tolerant to 2–6% salt concentration^29,30^. Another study reported that at salt concentrations above 6%, the bacterial density of *L. plantarum* ATCC 14917 decreased drastically^31^. Food products might be heated at various temperatures during food processing for safety and/or industrial purposes. Also, some food products contain higher amounts of salt. The fact that these rhizopsheric *L. plantarum* strains could survive at high temperatures and salt concentrations demonstrates their biotechnological potential for inclusion in such processed food products.

Before reaching the human gut, the orally ingested probiotics must overcome the effect of lysozyme which is present in the human saliva. Lysozyme disrupts the bacterial cell wall. Our results indicated that all the rhizosperic *L. plantarum* strains remained viable when exposed to lysozyme for 2 h, indicating their potential to reach the human GI tract in sufficient numbers. After reaching the human GI tract, the potential probiotics must sustain the effect of low pH and gastric juices present in the stomach. The pH of the stomach normally ranges between 2.5 and 3.5, although it might be as low as 1.5 during prolonged fasting or as high as 4.5 after a meal^32^. None of the *L. plantarum* strains survived at pH 1.5. However, the strain NS14 survived at both pH 2 and pH 3. All the *L. plantarum* strains remained viable in simulated gastric juice even after 2–3 h. Thus, the fact that *L. plantarum* strain NS14 could withstand assaults like acidic environment (pH 2–3) and simulated gastric juice reflects its potential to survive in the human stomach. An earlier study also reported about a probiotic strain*, L. plantarum* 9 which remained viable at acidic pH^33^.

Phenols are produced in the human GI tract as a result of deamination of aromatic aminoacids contained in the dietary proteins hence; phenol tolerance is also an essential attribute for survival of probiotics in the GI tract^34^. Both NS14 and NS16 could survive in culture medium containing 0.4% phenol for 24 h, while NGG could not (Table 6). Previous studies also indicated that among several *L. plantarum* strains tested for probiotic potential, only a few were phenol tolerant^28^. Another assault the potential probiotics need to overcome after their passage through the stomach is the action of bile juices (concentration ~ 0.3–0.5%) of the intestine^35^. Thus, the probiotic organisms should survive in the presence of bile salts. Though the number of bacterial colonies decreased slightly, all the *L. plantarum* strains remained viable in culture medium containing 0.5% bile salts even after after 2–4 h. Of all the *L. plantarum* strains tested, strain NS14 was found to be resistant to most of the clinically relevant antibiotics, except amoxicillin. Antibiotic resistance has been regarded as a beneficial probiotic property because a resistant probiotic when co-administered with an antibiotic might reduce the gastrointestinal side effects associated with the antibiotics^36^.

The ability to auto-aggregate and co-aggregate are important characteristics of probiotics because auto-aggregation ability helps them in adhering to mucosal surfaces and epithelial cells^37^ while co-aggregation ability helps them in preventing the colonization of human gut by pathogenic bacteria^38^. Both, *L. plantarum* strains NS14 and NGG exhibited a moderate capability of auto- and co-aggregation. Testing for hemolytic and DNase activities confirmed that all the *L. plantarum* strains might be safe for human consumption.

To summarize, our results indicated that all the rhizospheric *L. plantarum* isolates were capable of assimilating cholesterol in vitro, produced BSH, remained viable at high temperatures and in the presence of NaCl, lysozyme, simulated gastric juice, bile salts and, exhibited auto- and co-aggregation capabilities. Besides these attributes *L. plantarum* strain NS14 could survive in the presence of phenols, acidic environment (pH 2–3) and was resistant to many clinically relevant antibiotics. This suggests that it should be further investigated as potent probiotic in vivo with an additional benefit as a hypo-cholesterolemic biotherapeutic. Also, it can be stated that rhizosphere might be explored as a useful ecological niche for isolating *L. plantarum* strains which not only exhibit biotechnological potential, but also possess several essential and desirable probiotic attributes.

## Materials and methods

### Sample processing and isolation of LAB

Ten rhizospheric soil samples were collected from different parts of central India (Uttar Pradesh, Haridwar and New Delhi) following the standard microbial procedures and inoculated in screw capped plastic bottles containing 99 ml of De Man, Rogosa and Sharpe (MRS) broth. The methodology for sample processing and isolation were the same as described earlier^27^. Finally, three cultures that yielded pure colonies were presumed as LAB based on their growth in selective media like MRS broth and other characteristics like catalase negative test and Gram-positive staining. The glycerol stocks were prepared and preserved at − 80 °C which were revived at 37 °C prior to use.

### Identification of the isolates by sequencing of the gene encoding 16S rRNA

The three pure cultures that yielded pure colonies were designated in the laboratory as NS14, NS16 and NGG. The genomic DNA was extracted from the isolates using a commercial kit, mdi Genomic DNA Miniprep Kit (Ambala Cantt, India) and the gene encoding 16S rRNA was PCR-amplified using universal eubacterial primers (forward primer 5′AGAGTTTGATCCTGGCTCAG3′ and reverse primer 5′AGAGTTTGATCCTGGCTCAG3′). The contents of the PCR reaction mixture and the PCR reaction conditions were the same as reported earlier^27^. The PCR amplicons were purified using QIA Quick Gel Extraction Kit (Qiagen, Hilden, Germany) and sequenced using Sanger’s method at the Central Instrumentation Facility of University of Delhi South Campus, New Delhi. The homology in the gene sequences was determined using BLASTn.

### Temperature and sodium chloride tolerance

The ability of the strains to grow at different temperatures was investigated by growing them in MRS broth at 28, 37 and 45 °C. The capability to tolerate different concentrations of salt was investigated by inoculating them in MRS medium containing varying NaCl concentrations (0.5, 2, 3, 4, 5, 7 and 10%; w/v) at 37 °C. In either case, the absorbance of the inoculated and the control samples was recorded at 600 nm after 24 h. The experiment was repeated thrice for each isolate and the mean values were reported (± SD).

### Evaluation of probiotic characteristics

#### Tolerance to lysozyme and phenol

The tolerance of the strains to lysozyme was evaluated following a published method^39^. Briefly, overnight grown isolates were centrifuged, washed twice with PBS and suspended in Ringer solution. A 10 μl of this suspension was inoculated in a sterile electrolyte solution containing 100 mg/L lysozyme. The viable cell counts were determined for each strain after a time interval of 0.5, 1 and 2 h. For determining tolerance to phenol, overnight grown LAB strains were cultured in MRS broth containing 0.4% phenol at 37 °C, for 24 h. A 100 µl aliquot was removed at 0 h and 24 h and appropriate serial dilutions were made which were spread on the surface of MRS agar plates. These were further incubated at 37 °C and the number of colonies was determined after 24 h. Both the experiments were repeated thrice for each isolate and the mean values were reported (± SD).

#### Tolerance to acid and bile

The acid and the bile tolerance of the strains were evaluated following a published method^27^. The strains were inoculated in three separate flasks in which the pH of MRS medium ranged from 1.5, 2 and 3. After an interval of 1 h and 3 h, appropriate serial dilutions of 100 µl aliquots from each flask were inoculated on the surface MRS agar plates for 18–24 h, at 37 °C. The numbers of viable colonies were enumerated and results were expressed as log_10_ cfu/ml. The bile tolerance of *L. plantarum* strains was determined by inoculating them in MRS medium containing a commercial preparation of bile salts (sodium salts of cholic and deoxycholic acid; Sigma-Aldrich) at varying concentrations ranging from 0.125, 0.25, 0.5 and 1% (w/v), at 37 °C. Samples were withdrawn after 2 h and 4 h, 100 µl of appropriate serial dilutions were inoculated on MRS agar plates, viable cell colonies were enumerated and results were expressed as log_10_ cfu/ml. Both the experiments were repeated thrice for each isolate and the mean values were reported (± SD).

#### Tolerance to simulated gastric juice (pepsin)

The tolerance to simulated gastric juice (pepsin) was determined following a published method^27^. To prepare simulated gastric juice, 0.3% pepsin (w/v) was dissolved in 0.5% saline (v/v). The pH of this solution was adjusted to 3.0. Overnight grown *L. plantarum* strains were collected by centrifugation and the washed bacterial cells were suspended in 4 ml of sterile NaCl (0.8%) solution. Nine ml of the simulated gastric juice (pH 3) was mixed with 1 ml of the bacterial suspension. The cultures were kept at 37 °C in an incubator at 200 rpm. The viable cell counts were determined after 0, 2 and 4 h and the results were expressed as log_10_ cfu/ml. The experiment was repeated thrice for each strain and the mean values were reported (± SD).

### Auto-aggregation and co-aggregation

The ability of *L. plantarum* strains for auto-aggregation was determined following a published method^40^. Briefly, the *L. plantarum* strains were grown in MRS broth overnight and the cells were collected by centrifugation. The bacterial cells were washed and re-suspended in PBS and the cell density was adjusted to an absorbance of 0.5 at 600 nm. The suspension was kept for 2 h at 37 °C and the absorbance of the 1 ml of the upper phase was measured at 600 nm. The percentage cell auto aggregation was calculated as the percentage decrease in initial and final absorbance of the bacterial suspension relative to the initial absorbance of the bacterial suspension. The potential of *L. plantarum* strains to co-aggregate was determined by measuring the percentage reduction in absorbance of the mixed suspensions (*Escherichia coli* strain NG9 + *L. plantarum* strains) relative to the absorbance of the individual suspension (*L. plantarum* strain only) following a published method^41^.

### Bile salt hydrolase (BSH) activity and *bsh* gene

The BSH activity of *L. plantarum* strains was evaluated using a published method^27^. A sterile cork borer was used to punch wells of 6-mm diameter in MRS plates supplemented with a commercial preparation of 0.5% bile salts (sodium salt of cholic and deoxycholic acid; Sigma-Aldrich, United States). Then 200 µl of overnight MRS broth grown cultures were inoculated into these wells and the plates were incubated for 24–48 h at 37 °C. The presence of white zones of precipitation (opaque halo with silvery shine) around the wells was considered as a positive reaction. MRS agar plates without bile salt supplementation were used as controls.

Primer-Blast was used to design primers for PCR-amplification of *bsh* gene. The sequences of the forward (BF) and reverse (BR) primers were 5′TTACTTCGGTAGAAATTTCG 3′ and 5′TGATCGTAATGGATAAGAAA 3′, respectively. The components of the PCR reaction mixture and conditions were the same as described earlier^42^, except the annealing temperature which was kept at 55 °C. The PCR amplicons were purified and sequenced using the same methods as used for gene sequencing of 16S rRNA. The gene homology was determined using BLASTn. The gene sequences of *bsh* were translated using ExPASy and aligned using Clustal Ω.

### Potential for cholesterol assimilation

The potential of the LAB strains to assimilate cholesterol in MRS broth with and without bile salts was measured using published methods^27,43^. Briefly, LAB strains were incubated at 37 °C for 20 h in MRS broth supplemented with sodium tauroglycocholate (6 mM) and cholesterol (70 μg/ml). One ml of the supernatant was mixed with 2 ml of ethanol (95%) and 1 ml KOH (33%) and, heated for 15 min at 37 °C. This was followed by addition of 3 ml of *n*-hexane and 2 ml of distilled water. The mixture was left at the room temperature undisturbed and the hexane layer was transferred to another test tube and heated at 90 °C. The resultant residue was dissolved in 2 ml of *o*-phthalaldehyde (5 mg/ml), followed by the addition of 500 μl of concentrated sulphuric acid. The absorbance of the inoculated and un-inoculated samples was measured after 15 min at 570 nm. The cholesterol assimilation potential of each strain was calculated by dividing the assimilated cholesterol (μg/ml) after 20 h by the initial amount of cholesterol (μg/ml) at 0 h. The experiments were repeated for each isolate in triplicate and the mean percentage of cholesterol assimilation was reported ± standard deviation (SD).

### Antibiotic susceptibilities

The susceptibilities of the strains for commonly used antibiotics were determined using the Kirby Bauer disk diffusion method. The antibiotic disks (HiMedia, India) which were used in this study were—amoxicillin (10 μg), kanamycin (30 μg), streptomycin (10 μg), gentamycin (10 μg), ciprofloxacin (5 μg), erythromycin (15 μg) and tetracycline (30 μg). Antibiotic disks were placed on the bacterial lawn spread over the surface of Muller–Hinton (MH) agar plates and incubated at 37 °C for 24 h. The diameter of zone of inhibition around each antibiotic disk was measured and antibiotic susceptibilities of the strains were interpreted according to the guidelines of the Clinical Laboratory Standards Institute^44^.

### Evaluation of safety—hemolytic and DNase activities

The hemolytic activity of *L. plantarum* strains was determined following a published method^45^. Overnight grown strains were streaked on the surface of blood agar plates (7% v/v sheep blood). The plates were incubated at 37 °C for 48–72 h for development of a zone of hemolysis around the colonies. For DNase activity, the isolates were streaked on the surface of DNase agar medium plates and incubated at 37 °C. Formation of clear and pinkish zone around the colonies was considered as indication of DNase production^46^.

### Statistical analysis

All the experiments were performed in triplicates and the mean values ± standard deviation (calculated using Microsoft Excel) were reported. The statistical significance was calculated using the Mann–Whitney U test and *p* value < 0.05 was considered as significant.
